# The impact of summer vacation on children’s obesogenic behaviors and body mass index: a natural experiment

**DOI:** 10.1186/s12966-020-01052-0

**Published:** 2020-11-26

**Authors:** R. Glenn Weaver, Bridget Armstrong, Ethan Hunt, Michael W. Beets, Keith Brazendale, R. Dugger, Gabrielle Turner-McGrievy, Russell R. Pate, Alberto Maydeu-Olivares, Brian Saelens, Shawn D. Youngstedt

**Affiliations:** 1 Department of Exercise Science, Arnold School of Public Health, University of South Carolina, Columbia, USA; 2 Department of Health Sciences, University of Central Florida, Orlando, Florida USA; 3 Department of Health Promotion, Education, and Behavior, University of South Carolina, Columbia, SC USA; 4 Department of Psychology, University of South Carolina, Columbia, SC USA; 5 Seattle Children’s Hospital, Center for Child Health Behavior and Development, Seattle, Washington USA; 6 Arizona State University, Edson College of Nursing and Health Innovation, Phoenix, AZ USA

**Keywords:** Obesity, Policy, Children

## Abstract

**Background:**

Children’s BMI gain accelerates during summer. The Structured Days Hypothesis posits that the lack of the school day during summer vacation negatively impacts children’s obesogenic behaviors (i.e., physical activity, screen time, diet, sleep). This natural experiment examined the impact of summer vacation on children’s obesogenic behaviors and body mass index (BMI).

**Methods:**

Elementary-aged children (*n* = 285, 5-12 years, 48.7% male, 57.4% African American) attending a year-round (*n* = 97) and two match-paired traditional schools (*n* = 188) in the United States participated in this study. Rather than taking a long break from school during the summer like traditional schools, year-round schools take shorter and more frequent breaks from school. This difference in school calendars allowed for obesogenic behaviors to be collected during three conditions: Condition 1) all children attend school, Condition 2) year-round children attend school while traditional children were on summer vacation, and Condition 3) summer vacation for all children. Changes in BMI z-score were collected for the corresponding school years and summers. Multi-level mixed effects regressions estimated obesogenic behaviors and monthly zBMI changes. It was hypothesized that children would experience unhealthy changes in obesogenic behaviors when entering summer vacation because the absence of the school day (i.e., Condition 1 vs. 2 for traditional school children and 2 vs. 3 for year-round school children).

**Results:**

From Condition 1 to 2 traditional school children experienced greater unhealthy changes in daily minutes sedentary (∆ = 24.2, 95CI = 10.2, 38.2), screen time minutes (∆ = 33.7, 95CI = 17.2, 50.3), sleep midpoint time (∆ = 73:43, 95CI = 65:33, 81:53), and sleep efficiency percentage (−∆ = 0.7, 95CI = -1.1, − 0.3) when compared to year-round school children. Alternatively, from Condition 2 to 3 year-round school children experienced greater unhealthy changes in daily minutes sedentary (∆ = 54.5, 95CI = 38.0, 70.9), light physical activity minutes (∆ = − 42.2, 95CI = -56.2, − 28.3) MVPA minutes (∆ = − 11.4, 95CI = -3.7, − 19.1), screen time minutes (∆ = 46.5, 95CI = 30.0, 63.0), and sleep midpoint time (∆ = 95:54, 95CI = 85:26, 106:22) when compared to traditional school children. Monthly zBMI gain accelerated during summer for traditional (∆ = 0.033 95CI = 0.019, 0.047) but not year-round school children (∆ = 0.004, 95CI = -0.014, 0.023).

**Conclusions:**

This study suggests that the lack of the school day during summer vacation negatively impacts sedentary behaviors, sleep timing, and screen time. Changes in sedentary behaviors, screen time, and sleep midpoint may contribute to accelerated summer BMI gain. Providing structured programming during summer vacation may positively impact these behaviors, and in turn, mitigate accelerated summer BMI gain.

**Trial registration:**

ClinicalTrials.gov Identifier: NCT03397940. Registered January 12th 2018.

## Introduction

A growing body of evidence indicates elementary aged children (5–12 years) in the United States experience accelerated gains in body mass index (BMI) during the summer [1–3]. Accelerated summer BMI gain has also been observed in Canada and Japan [4] and a recent study aims to examine this phenomenon in Australia [5]. The mechanisms driving accelerated BMI gain during summer are unclear and are likely due to a complex web of behavioral, environmental, and biological factors [6, 7]. From a behavioral perspective accelerated summer BMI gain may be due to increased engagement in unhealthy obesogenic behaviors (i.e., physical activity, sleep, screen time, and diet) during summer [6]. For example, during summer children may increase sedentary and screen time, reduce engagement in moderate-to-vigorous physical activity (MVPA) increase consumption of unhealthy foods, decrease consumption of healthy foods, and decrease sleep duration while shifting sleep timing later (i.e., going to sleep and waking later). All of these behaviors have been linked to increased risk for overweight or obesity [8–14]. Further, in the United States children typically do not attend school for up to 12 weeks during the summer (May–August), commonly referred to as summer vacation. Engagement in behaviors that are linked to increased risk for overweight or obesity during summer vacation may be directly linked to the absence of the school day during summer.

The structured days hypothesis (SDH) posits that structure, defined as a pre-planned, segmented, and adult-supervised compulsory environment, helps to minimize children’s engagement in undesirable obesogenic behaviors [6]. Thus, children may engage in less desirable levels of obesogenic behaviors during summer vacation because the compulsory, pre-planned, and adult supervised structure provided by the school day is no longer present on a consistent basis. Few studies have examined children’s obesogenic behaviors during summer vacation compared to the school year, however, studies that compare school to weekend days (i.e., typically less-structured) overwhelmingly support the SDH. Studies that have examined obesogenic behaviors in children during school compared to summer vacation are limited because they used between group designs, had limited samples sizes, and typically examined behaviors during summer vacation and the school year for 1 week or less [15–22]. Previous studies have also failed to consider that children’s behaviors naturally fluctuate seasonally. In cooler climates studies have shown that physical activity increases during the summer when days are milder compared to the winter [23, 24], while in warmer climates physical activity may decrease during the summer due to extreme heat and humidity [25, 26]. In addition, more recent studies have suggested that extended exposure to daylight and artificial lighting during the afternoons and evenings in the summer may lead to delayed circadian timing resulting in shifts to later sleep timing [7, 27].

**Fig. 1:**
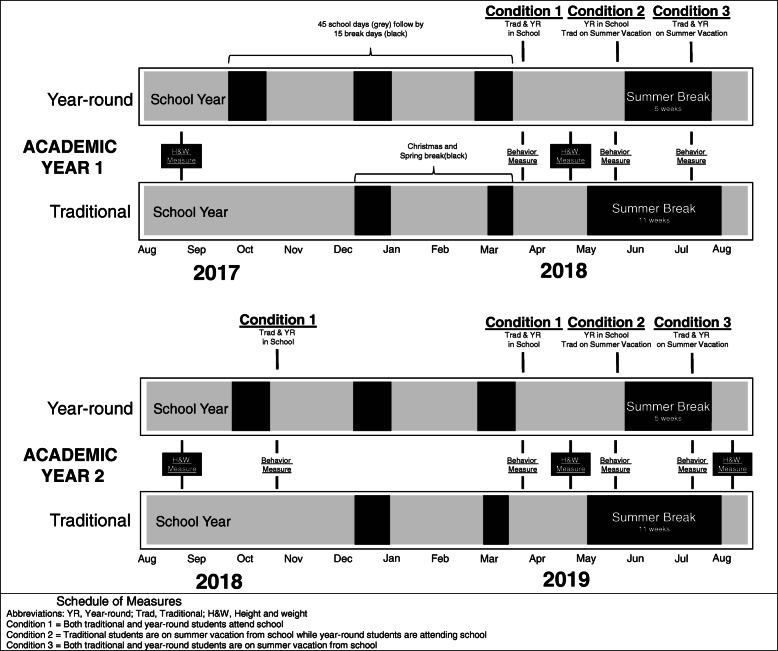
Schedule of Measures. Abbreviations: YR, Year-round; Trad, Traditional; H&W, Height and weight. Condition 1 = Both traditional and year-round students attend school. Condition 2 = Traditional students are on summer vacation from school while year-round students are attending school. Condition 3 = Both traditional and year-round students are on summer vacation from school

Year-round schools (aka distributed calendars) operate on a 180-day schedule similar to traditional schools. However, year-round schools incorporate shorter, more frequent breaks throughout the calendar year rather than taking one prolonged 2–3-month break over summer. A typical year-round school operates for 9 weeks in a row and then takes a 3-week break from school. Year-round school schedules are relevant when examining changes in children’s obesogenic behaviors during summer vacation as children attending year-round schools are exposed to school during the traditional summer vacation. Thus, comparing behaviors of children in traditional and year-round schools when both schools are in session (Condition 1), traditional schools are on summer vacation but year-round schools are in session (Condition 2), and both schools are on summer vacation (Condition 3—see Fig. 1) allows one to disentangle naturally occurring seasonal fluctuations in children’s obesogenic behaviors from changes that occur due to the absence of the school day during summer vacation. To the author’s knowledge no previous studies have capitalized on differing school calendars to test the SDH.

The purpose of this study was to examine the impact of summer vacation on elementary-aged children’s obesogenic behaviors during the summer using a natural experiment design. Participants in the study attended schools following either a traditional or year-round school calendar. Using a school following a year-round calendar as a seasonal control we aimed to examine if changes in traditional school children’s behavior are due to the removal of the school day not due to changing seasons. To accomplish this the following hypotheses were tested.

### Hypotheses

1. Children attending the traditional school will experience larger unhealthy changes in behaviors (physical activity, screen time, diet, and sleep) than year-round school children from Condition 1 to Condition 2 (i.e., when traditional school children stop attending school but year-round school children continue attending school).
2. Children attending the year-round school will experience greater unhealthy changes in behaviors than traditional school children from Condition 2 to Condition 3 (i.e., when year-round school children stop attending school and traditional school children remain on summer vacation).

## Methods

### Setting and participants

This study is part of a larger natural experiment that aims to examine changes in BMI and fitness during the traditional summer vacation and during the school year for children attending a year-round school and two match paired traditional schools [28]. Three schools in one urban school district in the southeastern United States participated in the current study. One school (i.e., school A) converted to a year-round schedule in the fall of 2016. The year-round calendar called for children to attend school for 9 weeks and then to take a 3-week break from school. During June and July, the traditional summer vacation, the year-round school took an extended 5-week break. This school is the only school in the school district following a year-round calendar. With the exception of the year-round calendar, the school follows all district policies and procedures, including school zoning practices (i.e., how children are assigned to attend specific schools within the district). Specifically, the decision for children to attend the year-round school is made by the district, not families, and based on home address. Two match-paired schools (i.e., schools B and C) were selected to participate because of similar school day structure, daily start and end times, student race/ethnicity, gender, number of students enrolled, age/grade levels served, percentage of students receiving free and reduced lunch, and academic test scores. Table 1 presents the demographics of the participating schools and individual participants. Table 2 displays the flow of participants throughout the study.

**Table 1:** Characteristics of participant schools and students

	A Year-round Calendar	B Traditional Calendar	C Traditional Calendar	All Schools
Participating School Characteristics				
Total Students	389	443	417	1249
Male (%)	49.4	60.5	50.4	53.6
Age in Years	4–12 years	4–12 years	5–12 years	4–12 years
Grades	prek-6	prek-6	k-6	prek-6
Race				
White (%)	28.8	23.5	34.3	28.7
Black (%)	60.9	67.0	55.9	61.4
Other Race/ethnicity (%)	10.3	8.5	8.8	8.8
Free & Reduced Priced Lunch (%)	81.0	87.0	84.0	84.0
Current Study Participant Characteristics at Baseline				
Number of participants	97	93	95	285 ^a^
Male (%)	59.2	44.2	41.5	48.7
Age in Years (SD)	7.3 (1.3)	7.6 (1.3)	7.2 (1.2)	7.4 (1.3)
Grade (SD)	2.4 (1.2)	2.4 (1.2)	2.2 (1.1)	2.3 (1.1)
BMI z-score (SD)	0.56 (1.14)	0.59 (1.40)	0.66 (1.21)	0.61 (1.23)
Household Income				
< 40,000 (%)	55.9	60.0	55.3	57.0
≥ 40,000 (%)	44.1	40.0	44.7	43.0
Race				
White (%)	24.7	19.6	42.4	29.5
Black (%)	59.0	78.4	39.4	57.4
Other Race/ethnicity (%)	6.9	0.0	13.6	7.4
Not reported (%)	9.8	2.0	4.6	5.8
Mean Number of Measurement Waves with Valid Data* (range 1–4)*	2.9	2.4	2.6	2.6
Mean Number of Valid Days of Data				
Physical Activity (SD)^b^	70.4 (31.4)	50.5 (33.5)	68.8 (39.0)	63.6 (31.0)
Sleep (SD) ^b^	35.4 (26.0)	24.6 (30.3)	32.5 (23.0)	30.9 (26.2)
Foods Consumed & Screen Time (SD) ^c^	26.8 (20.5)	20.3 (19.3)	23.4 (20.4)	23.6 (20.1)

^a^ Includes total number of unique children providing valid data from the original and refresh cohort

^b^ Maximum of 142 wear days

^c^ Maximum of 41 possible daily diary reports

**Table 2:** Flow of participants through measurement waves

		Year 1				Year 2			
		Recruited Feb 2018	Condition 1 Mar 2018	Condition 2 May, Jun, & Jul 2018	Condition 3 Jul & Aug 2018	Recruited Aug & Sep 2019	Condition 1 Mar 2019	Condition 2 May, Jun, and Jul 2019	Condition 3 Jul & Aug 2019
Original Cohort	Traditional (n)	160	117	100	89	–	78	69	69
	Year-Round (n)	80	75	62	60	–	49	43	41
	Total (n)	240	192	162	149	–	127	112	110
Refresh	Traditional (n)	–	–	–	–	71	52	52	50
	Year-Round (n)	–	–	–	–	20	15	15	13
	Total (n)	–	–	–	–	91	67	67	63
Grand Total (n)		240	192	162	149	240	194	179	173

Grand total represents sum of the number of participants in the original cohort and the refresh cohort

Condition 1 = Both traditional and year-round students attend school

Condition 2 = Traditional students are on summer vacation from school while year-round students are attending school

Condition 3 = Both traditional and year-round students are on summer vacation from school

### Power analysis

An a priori power analysis for the smallest detectable effect was performed using G*Power (v.3.1.9.2) and was based on the difference in change of behaviors between groups. With a total of 240 children (using a variance inflation correction factor of 1.74 to account for clustering within grades per school) [29], and according to the statistical software G*power 3.1.9.7, the study is sufficiently powered to detect a difference between intervention groups of *d* = 0.23 with a power = 80% and α = 0.05. This was determined to be sufficient power as previous studies that have examined changes in obesogenic behaviors during the summer have found Cohen’s *d* effects of 0.21 (i.e., diet) to 0.78 (i.e., physical activity) [15–22, 30].

### Procedures

Behavioral data were collected on a subset of children participating in the larger study from Spring 2018-Fall 2019. This study presents the behavioral data in addition to changes in BMI during the corresponding school years (2017–2018, 2018–2019) and summers (2018, 2019). All kindergarten through third grade (i.e., 5–8 years) students participating in the larger study were invited to participate in the behavioral data collection in the Spring of 2018. A consent letter was sent home to the parents describing the study procedures. Parents who consented to their child’s participation were asked to sign and return the letter to the school where it was retrieved by research staff. From the 254 children whose parents consented a total of 240 were randomly selected to participate in the study. Measurements commenced in the spring academic semester of 2018 (i.e., March) and were completed in the fall academic semester of 2019 (i.e., August). In the fall of 2018 a refreshment sample was recruited to replace children who dropped out of the study (e.g., did not complete behavioral measures, family moved). The refreshment sample was recruited using the same procedures described for the original sample at the participating schools and were matched on age, sex, and race/ethnicity of the children they replaced. Table 2 presents data on the number of children recruited to participate in the original sample and for the refreshment sample. Data were collected during three distinct conditions: Condition 1 when both traditional and year-round school children were attending school, Condition 2 when traditional children were on summer vacation, but year-round children were attending school, and Condition 3 when both traditional and year-round school children were on summer vacation. Figure 1 depicts the schedule of measurements. Condition 1 was collected during March of 2018 and 2019. Each measurement period for Condition 1 lasted approximately 1 month. Condition 2 and 3 were collected during late May, June, July, and early August of 2018 and 2019. Data during these conditions were collected during one extended measurement period lasting approximately 3 months (Condition 2 lasts 6 weeks, Condition 3 lasts 5 weeks). All protocols were approved by the lead author’s University Institutional Review Board.

### Measures

#### Physical activity and sleep

As described previously [31], physical activity and sleep were measured using a Fitbit Charge 2™ (Fitbit Inc., San Francisco, California, USA). Fitbits were chosen because Fitbit Charge devices have be shown to provide sleep and heartrate estimates in elementary aged school children and adolescents that demonstrate good agreement with polysomnography and electrocardiography, respectively [32, 33], and wrist-worn scientific grade devices used to assess free-living sleep [34]. Further, because participants can charge Fitbit devices at home and data is stored in the cloud Fitbits allowed for data collection over extended periods of time (e.g., 3-month summer vacation) without the need to replace devices due to battery or data storage limitations. Data processing was informed by the ISCOLE data processing protocols [35].

Fitbit sleep data were exported to identify child sleep episodes. For this study sleep duration, timing and quality were considered as they have all be linked to risk for overweight or obesity [11, 14]. For sleep timing, sleep onset was defined as the first minute that a sleep episode began. Sleep offset was defined as the last minute that a sleep episode was recorded. Sleep midpoint was calculated by identifying the time halfway between sleep onset and sleep offset. Sleep midpoint is a common indicator of shifts in sleep timing in sleep research as it takes into account both sleep onset and offset [36, 37]. For duration, total sleep time was identified as the number of minutes that the Fitbit device classified a child as asleep during a sleep episode. For quality, sleep efficiency was calculated by dividing the total sleep time by time in bed. For this paper, only nocturnal sleep was considered. Nocturnal sleep was defined as sleep onset times that occurred between 5 pm and 6 am and lasted for greater than 240 min [38]. If sleep segments were separated by less than 20 min they were considered one continuous sleep segment [35].

To distill the heartrate data into activity intensity levels, each child’s resting heartrate was identified as the lowest mean beats-per-minute for 10 consecutive minutes each day [39–42]. Resting heartrates were calculated for each child each wear day. Heartrate has been widely used to determine activity intensity in children [43]. Heartrates were distilled into activity intensity levels based on percent heart rate reserve (HRR). That is, 0.0–19.9% of HRR equaled sedentary, 20.0–49.9% of HRR equaled light physical activity, and ≥ 50.0% equaled MVPA [44, 45]. Sleep episode data were mapped onto a child’s physical activity data to determine sleep and wake times. A day with at least 10 h of waking wear was considered as a valid day of wear [35]. Valid days were distilled into total waking time children spent sedentary and in MVPA on each day.

#### Healthy foods, unhealthy foods, and screen time

Children’s consumption of healthy and unhealthy foods and screen time were assessed via parent proxy-report. Parents received a daily diary via text message which asked them to report their child’s screen time and foods consumed twice per week. Parents were asked to report on their child’s screen time and foods on at least 4 days during each measurement condition (i.e. Condition 1–3). Parents were encouraged to complete the diaries along with their child to enhance the accuracy of the estimates. Parents/children estimated the total amount of time (hours and minutes) spent in front of a screen that day (e.g., TV, computer, video game, smartphone, and tablet) [46, 47]. Similar to past studies [22, 48], healthy and unhealthy foods were assessed using the Beverage and Snack Questionnaire [49]. Items were scored by four possible response categories: 0 (‘child did not consume’), 1(‘child consumed a little’), 2(‘child consumed some’), and 3 (‘child consumed a lot’) with those individual items. For this study, individual food items were grouped in accordance with the Healthy Meal Index [50]. Food categories included: fruits, vegetables, dairy (non-sugar sweetened), convenience foods, sweets and desserts, and sugar sweetened beverages (including dairy), water. Two groups were created for analysis of foods consumed: healthy foods/drinks (fruits, vegetables, and unsweetened dairy, water), and unhealthy foods/drinks (convenience foods, and sweets/desserts, sugar sweetened beverages). Consumption was dichotomized (i.e., ‘did’ vs. ‘did not’ consume) and reported as mean days per week that a healthy or unhealthy food/drink was consumed [49].

#### Body mass index

Changes in children’s heights and weights were measured for the 2017/18 school year (August 2017–May 2018), the 2018 summer (May–August 2018), the 2018/19 school year (August 2018–May 2019), and the 2019 summer (May–August 2019). All measures in both the year-round and traditional schools were based upon the traditional school calendar and occurred during the same two-week period. Measures were completed during the last (end of school year) or first (beginning of school year) 2 weeks of the traditional school year. All measures were obtained during regularly scheduled physical education class. Using a portable stadiometer (Model S100, Ayrton Corp., Prior Lake, Minn.) and digital scale (Healthometer model 500KL, Health o meter, McCook, Ill.), children’s heights (nearest 0.1 cm) and weights (nearest 0.01 lbs.), without shoes, were collected by research assistants. BMI was calculated (BMI = kg/m^2^) and transformed into age and sex specific z-scores (zBMI) [51].

### Statistical analyses

All analyses were completed in Stata (v14.2, College Station, TX) during April of 2019. Prior to completing the primary analyses descriptive means and standard deviations of school and child characteristics were examined. To examine the differences in obesogenic behaviors during the school year and summer break from school, multi-level mixed effect linear regressions, with days nested within children, were estimated. Separate models were estimated for each variable related to the four measured obesogenic behaviors including [1] sedentary time, [2] light physical activity, [3] MVPA, [4] screen time, [5] unhealthy foods index, [6] healthy foods index, [7] total sleep time, [8] sleep midpoint, and [9] sleep efficiency. A three-level condition (0 = Condition 1, 1 = Condition 2, 3 = Condition 3) and two-level school calendar (i.e., 0 = traditional or 1 = year-round) variable were considered the independent variables. A condition by school calendar interaction was also included to test our hypotheses that behaviors would change differently between groups from one condition to the next. From Condition 1 to 2 the interaction was calculated by subtracting year-round from traditional. From Condition 2 to 3 the interaction was calculated by subtracting traditional from year-round. In order to ensure that comparisons were between school days and summer break days, weekend days and school break days (i.e., spring break, teacher workdays) were excluded from all models.

Monthly zBMI change was also examined among the participating children for the corresponding school and summers using multi-level mixed effect linear regressions, with repeated measures nested within children. Monthly zBMI change was the dependent variable with a two-level condition (school vs. summer), two-level school calendar (traditional vs. year-round) variable, and the condition-by-school calendar interaction as the independent variables. Monthly zBMI change was used to standardize change during the summer (i.e., 3 months) and school year (i.e., 9 months).

All statistical models included sex, race/ethnicity, grade, academic year, and refreshment status (original vs. refresh participants) as covariates. Physical activity models included wear time as an additional covariate. The Benjamini-Hochenberg procedure with a false discovery rate of 10% was utilized to account for multiple comparisons [52].

## Results

Characteristics of the participating schools and students are presented in Table 1. Table 2 displays the flow of participants through the study. Table 3 presents the means and standard deviations of each obesogenic behavior, by school calendar type, during each measurement condition.

**Table 3:** Raw daily estimates of obesogenic behaviors by group and condition

			Condition 1				Condition 2				Condition 3			
Construct	Behavior	School Type	Mean	(SD)	Difference	*p*-value^a^	Mean	(SD)	Difference	*p*-value^a^	Mean	(SD)	Difference	*p*-value^a^
Physical Activity	Sedentary minutes	Traditional	367.3	(227.3)	−25.3	0.81	395	(263.9)	−13.5	0.09	394.2	(250.7)	−48.4	0.11
		Year-round	392.6	(227.5)			408.5	(227.2)			442.6	(247.2)		
	LPA Minutes	Traditional	505.1	(159.7)	12.7	0.83	491.7	(157.8)	1.1	0.15	− 483.5	(147.7)	−50.6	0.13
		Year-round	492.4	(151.7)			490.6	(163.5)			− 432.9	(146.1)		
	MVPA minutes	Traditional	62.5	(50.3)	−2.1	0.24	84.5	(81.3)	2.4	0.24	91.6	(102.7)	16.9	0.44
		Year-round	64.6	(54.8)			82.1	(98.3)			74.7	(79.2)		
Screen Time	Screen Time minutes	Traditional	92.6	(85.7)	−11	0.30	134.8	(114.9)	18.5	0.09	138.2	(107.8)	**−28.6**	**0.04**
		Year-round	103.6	(84.4)			116.3	(101.1)			166.8	(133.9)		
Diet	Unhealthy Foods	Traditional	2.72	(1.76)	−0.25	0.52	2.96	(1.77)	−0.14	0.23	3.03	(1.76)	−0.02	0.79
		Year-round	2.97	(1.79)			3.1	(1.86)			3.05	(1.68)		
	Healthy Foods	Traditional	1.47	(1.04)	−0.08	0.54	1.52	(1.07)	0.1	0.52	1.54	(1.04)	0.2	0.11
		Year-round	1.55	(1.05)			1.42	(1.06)			1.34	(1.03)		
Sleep	Sleep minutes	Traditional	467.7	(67.6)	−4.9	0.44	486	(92.3)	17.5	0.17	489.9	(87.6)	3.4	0.55
		Year-round	472.6	(69.6)			468.5	(69.5)			486.5	(94.0)		
	Sleep Onset	Traditional	22:03:15	(72:26)	0:49	0.58	23:08:20	(104:43)	**0:54:49**	**< 0.00**	23:23:20	(103:09)	−0:28:19	0.06
		Year-round	22:02:26	(71:27)			22:13:19	(73:51)			23:51:39	(104:15)		
	Sleep Offset	Traditional	6:22:45	(39:13)	−1:51	0.94	7:53:19	(94:18)	**1:20:00**	**< 0.00**	8:11:40	(99:29)	**−0:21:39**	**0.00**
		Year-round	6:24:36	(43:11)			6:33:19	(55:28)			8:33:19	(103:15)		
	Sleep Midpoint	Traditional	2:12:32	(46:39)	−1:24	0.78	3:33:20	(98:35)	**1:10:04**	**< 0.00**	3:48:20	(90:38)	**−0:23:20**	**0.01**
		Year-round	2:13:56	(46:36)			2:23:16	(53:41)			4:11:40	(90:39)		
	Sleep Efficiency	Traditional	93.9	(5.2)	−0.3	0.76	92.8	(6.7)	−1.1	0.15	93	(6.7)	−0.4	0.58
		Year-round	94.2	(3.2)			93.9	(3.1)			93.4	(3.0)		

^a^Based on multilevel mixed effects linear regression

Bolded text represents statistically significant point estimates at Benjamini-Hochberg critical value of ≤0.05 with a 10% false discovery rate

Abbreviations *“MVPA”* Moderate to vigorous physical activity, *“LPA”* Light physical activity

Condition 1 = Both traditional and year-round students attend school

Condition 2 = Traditional students are on summer vacation from school while year-round students are attending school

Condition 3 = Both traditional and year-round students are on summer vacation from school

### Hypothesis 1

Children attending the traditional school will experience larger unhealthy changes in behaviors (physical activity, screen time, diet, and sleep) than year-round school children from Condition 1 to Condition 2.

Findings related to hypothesis 1 can be found in Table 4. Consistent with Hypothesis 1 children attending the traditional school experienced statistically significant greater increases in sedentary minutes (∆ = 24.2, 95CI = 10.2, 38.2), screen time (∆ = 33.7, 95CI = 17.2, 50.3), and sleep midpoint (∆ = 73:43, 95CI = 65:33, 81:53) from Condition 1 to Condition 2 when compared to year-round school children. Also consistent with Hypothesis 1 sleep efficiency (∆ = − 0.7, 95CI = -1.1, − 0.3) and light physical activity (∆ = − 23.4, 95CI = -35.3, − 11.6) decreased to a greater degree among children in traditional schools. Contrary to the hypothesis sleep duration (∆ = 16.1, 95CI = 6.5, 25.6) increased more in traditional when compared to the year-round school children from Condition 1 to Condition 2. No other differences in change reached statistical significance.

**Table 4:** Model implied within group change and difference in change in daily estimates of obesogenic behaviors between groups

Construct	Behavior		Condition 1 vs. Condition 2						Condition 2 vs. Condition 3					
			Within Group			Between Group^a^			Within Group			Between Group^b^		
			Change	95CI		Difference in Change	95CI		Change	95CI		Difference in Change	95CI	
Physical Activity	Sedentary minutes	Traditional	**35.1**	**(26.0,**	**44.2)**	**24.2**	**(10.2,**	**38.2)**	7.8	(−3.0,	18.7)			
		Year-round	**10.9**	**(0.2,**	**21.6)**				**62.3**	**(49.9,**	**74.7)**	**54.5**	**(38.0,**	**70.9)**
	LPA minutes	Traditional	**−36.0**	**(−43.7,**	**−28.3)**	**−23.4**	**(−35.3,**	**−11.6)**	**−13.1**	**(−22.3,**	**−4.0)**			
		Year-Round	**−12.6**	**(−21.6,**	**−3.6)**				**−55.4**	**(−65.9,**	**−44.9)**	**− 42.2**	**(−56.2,**	**−28.3)**
	MVPA minutes	Traditional	0.9	(−3.3,	5.2)	−0.6	(−7.2,	5.9)	5.1	(−0.1,	10.1)			
		Year-round	1.6	(−3.4,	6.6)				**−6.3**	**(−12.2,**	**−0.5)**	**−11.4**	**(−19.1,**	**− 13.7)**
Screen Time	Screen Time minutes	Traditional	**50.1**	**(39.8,**	**60.5)**	**33.7**	**(17.2,**	**50.3)**	−0.1	(−10.5,	10.3)			
		Year-round	**16.4**	**(3.5,**	**29.3)**				**46.4**	**(33.7,**	**59.2)**	**46.5**	**(30.0,**	**63.0)**
Diet	Unhealthy Foods Index	Traditional	**0.18**	**(0.00,**	**0.35)**	0.08	(−0.20,	0.36)	0.09	(−0.09,	0.27)			
		Year-round	0.10	(−0.12,	0.32)				0.09	(−0.13,	0.30)	0.00	(−0.28,	0.28)
	Healthy Foods Index	Traditional	0.01	(−0.09,	0.11)	0.16	(−0.01,	0.32)	0.05	(−0.05,	0.15)			
		Year-round	**−0.15**	**(−0.27,**	**− 0.02)**				−0.05	(−0.18,	0.07)	−0.11	(− 0.27,	−0.06)
Sleep	Sleep min	Traditional	**12.7**	**(6.6,**	**18.9)**	**16.1**	**(6.5,**	**25.6)**	3.9	(−4.0,	11.8)			
		Year-round	−3.3	(−10.6,	4.0)				**19.5**	**(9.8,**	**29.1)**	**15.6**	**(3.1,**	**28.0)**
	Sleep Midpoint Time	Traditional	**85:03**	**(79:49,**	**90:17)**	**73:43**	**(65:33,**	**81:53)**	**17:24**	**(10:46,**	**24:02)**			
		Year-round	**11:19**	**(5:03,**	**17:36)**				**112:19**	**(104:12,**	**120:25)**	**95:54**	**(85:26,**	**106:22)**
	Sleep Efficiency %	Traditional	**−0.8**	**(−1.0,**	**−0.5)**	**−0.7**	**(−1.1,**	**−0.3)**	0.2	(−0.2,	0.5)			
		Year-round	−0.1	(−0.4,	0.2)				−0.3	(−0.7,	0.1)	0.5	(−1.0,	0.0)

Abbreviations *“MVPA”* Moderate to vigorous physical activity, *“LPA”* Light physical activity

Bolded text represents statistically significant point estimates at Benjamini-Hochberg critical value of ≤0.05 with a 10% false discovery rate

^a^ Interaction is calculated by subtracting year-round from traditional

^b^ Interaction is calculated by subtracting traditional from year-round

Condition 1 = Both traditional and year-round students attend school

Condition 2 = Traditional students are on summer vacation from school while year-round students are attending school

Condition 3 = Both traditional and year-round students are on summer vacation from school

### Hypothesis 2

Children attending the year-round school will experience greater unhealthy changes in behaviors than traditional school children from Condition 2 to Condition 3.

Findings related to hypothesis 2 can also be found in Table 4. Consistent with hypothesis 2 children attending the year-round school experienced statistically significant greater increases in sedentary minutes (∆ = 54.5, 95CI = 38.0, 70.9), screen time (∆ = 46.5, 95CI = 30.0, 63.0), and sleep midpoint (∆ = 95:54, 95CI = 85:26, 106:22) from Condition 2 to Condition 3 when compared to traditional school children. Also consistent with hypothesis 2 year-round school children experienced statistically significant greater declines in light physical activity (∆ = − 42.2, 95CI = -56.2, − 28.3) and MVPA (∆ = − 11.4, 95CI = -19.1, − 3.7) relative to traditional school children. This difference in change was driven by a larger statistically significant decline in year-round school children’s light physical activity (∆ = − 55.4, 95CI = --65.9, − 44.9) and MVPA (∆ = − 6.3, 95CI = -12.2, − 0.5) from Condition 2 to 3. Contrary to the hypothesis children in the year-round school increased sleep min (∆ = 15.6, 95CI = 3.1, 28.0) to a greater degree than the traditional school children. No other differences in change reached statistical significance.

### Changes in children’s zBMI

**Fig. 2:**
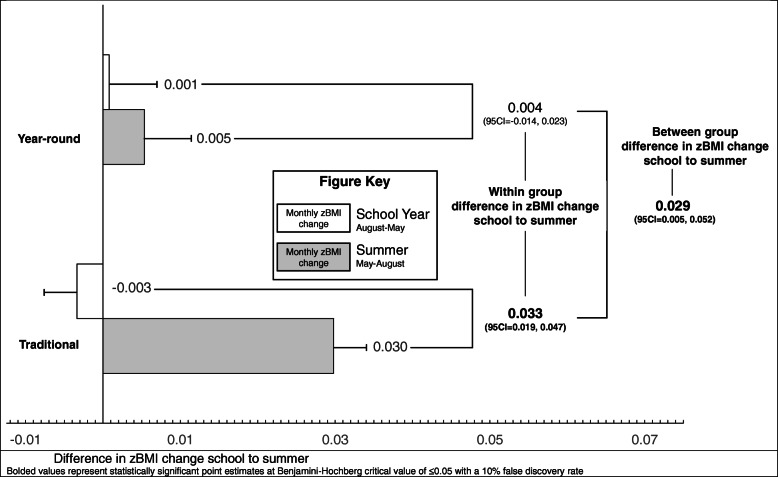
Difference in zBMI change school to summer. Bolded values represent statistically significant point estimates at Benjamini-Hochberg critical value of ≤0.05 with a 10% false discovery rate

Changes in children’s zBMI are presented in Fig. 2. Children in the traditional school experienced a monthly change in zBMI of − 0.003 and 0.030 during the school year and summer, respectively. This translated to a statistically significant difference in change between school year and summer of 0.033 (95CI = 0.019, 0.047). Children in the year-round school experienced a monthly change in zBMI of 0.001 and 0.005 during the school year and summer, respectively. This translated to a difference in change between school year and summer of 0.004 (95CI = -0.014, 0.023). The difference in school to summer change in zBMI between year-round and traditional schools was 0.029 (95CI = 0.005, 0.052).

## Discussion

This natural experiment examined the impact of summer vacation (i.e., the absence of the school day during summer) on children’s obesogenic behaviors. The findings suggest that the absence of the school day during summer vacation increases children’s engagement in sedentary behaviors and screen time while shifting children’s sleep later. However, it appears that children sleep more during summer vacation. Findings from this study related to children’s engagement in MVPA, dietary behaviors, and sleep efficiency were mixed. Corresponding accelerations in zBMI gain were observed during the summer for traditional but not year-round school children. Identifying changes in obesogenic behaviors during summer vacation is of particular importance as a growing body of evidence indicates that children around the world are at risk for accelerated BMI gain during this time [1–3]. Further, accelerations in BMI gain during summer were previously observed in children attending schools following a traditional calendar in this study [28].

This study found that traditional school children shifted sleep timing more than 1-h later from Condition 1 to 2 (i.e., when summer vacation began) and that this shift was statistically significantly greater than the approximate 10-min shift experienced by year-round school children during the same measurement wave (i.e., when year-round school children were still attending school). Further, children in the year-round school shifted sleep timing by almost 2 h from Condition 2 to 3 (i.e., when summer vacation began for year-round school children) and traditional school children only saw an approximate 20-min shift over the same timeframe (i.e., traditional school children remained on summer vacation from Condition 2 to 3). Shifts to longer sleep duration were also observed for children in the traditional and year-round schools when summer vacation began. Traditional school children also saw a decrease in sleep quality when summer vacation began but year-round school children did not. These findings indicate that children may naturally shift sleep timing later during the summer by approximately 10–20 min. This shift in sleep timing did not appear to be accompanied by a shift to longer sleep duration. However, the absence of school appears to impact sleep timing and duration with timing shifting 1- to 2-h later and duration increasing by approximately 15 min.

This finding is consistent with the SDH and previous studies that have shown that on weekends, during school breaks (i.e., spring break), and summer vacation children sleep longer [16, 17, 22] and go to bed and wake later [17, 31, 53]. Shifting sleep later and sleeping for longer on days away from school is commonly referred to as social jetlag [53]. Longer sleep during days away from school may be due to children compensating for missed sleep during school days. Thus, from a social jetlag perspective, days away from school represent natural and healthier sleep patterns. However, later sleep timing (i.e., later bed and wake times) has also been associated with increased risk for overweight and obesity in elementary aged children and adolescents, even when controlling for sleep duration [14, 54–56]. The reason for this relationship remains unclear but at least one study in adolescents found that later bed times were associated with increased daily energy intake and screen use [57]. Later wake times may also increase the likelihood that children and adolescents skip breakfast, which has been associated with increased risk for overweight and obesity in elementary aged children [58] and adolescents [59]. Shifts in meal timing have also recently been identified as an indicator of risk for overweight or obesity [60]. Whatever the mechanism, the finding that children are sleeping later and longer during summer vacation coupled with data indicating that BMI gain accelerates as well suggests that even though children are sleeping more during summer vacation, later sleep timing – and any co-existing unhealthy behaviors (e.g., evening snacking), may be overriding the benefits derived from increased sleep duration.

This study also found that traditional school children increased parent proxy-reported screen time and objectively measured sedentary minutes by more than 30 min during summer vacation and that this shift was statistically significantly greater than the approximate 10- to 15-min increase in screen time and sedentary minutes experienced by year-round school children who were still attending school during the same measurement wave. Further, children in the year-round school increased screen time and sedentary minutes by more than 45 min when the year-round school was not in session during summer vacation. Traditional school children only saw approximately a 10-min increase in sedentary minutes with no increase in screen time over the same timeframe. The finding that daily screen time and sedentary minutes increased for both traditional and year-round school children during summer vacation provides causal evidence that the school day regulates these behaviors. This finding is also consistent with the SDH and other studies that show children increased sedentary activities during days away from school [6].

Evidence for changes in MVPA were mixed. Children in the traditional school did not experience decreases in MVPA during summer vacation while children in the year-round school experienced an approximate 6-min reduction in MVPA. It is unclear why sedentary would be impacted but not MVPA. Regardless this finding suggests that reducing sedentary time during summer vacation may be a target for intervention to mitigate accelerated summer BMI gain.

This study compliments and extends the evidence of the few studies that have examined differences in obesogenic behaviors between the summer break and school year. To our knowledge only three studies have examined within-participant differences in obesogenic behaviors between the school year and summer vacation. Even though these studies were relatively small, ranging from 14 to 89 participants, all found that children increased time sedentary over summer vacation [21, 22, 61], consistent with the current study. The two studies that examined changes in diet during the summer vacation produced mixed findings [22, 61]. One study found that children ate more fruit during the summer vacation [22] while the other found the opposite [61]. The current study found that changes in consumption of healthy and unhealthy foods were minimal and not statistically significant. This is possibly due to the use of parental report and food frequency questionnaires in the current study. Food frequency questionnaires may not be sufficiently sensitive to capture changes in children’s diet between structured and less structured days. Additionally, parents may be less aware of the foods children eat on days that they attend school. Multiple pass 24-h dietary recall, the gold standard of free-living dietary assessment [62] may be necessary in future studies. Thus, the impact of the school day on diet is unclear. Finally, one study examined changes in sleep and screen time during the summer vacation [22]. Consistent with the current study, both screen time and sleep duration increased.

Corresponding with some of the observed behavioral changes, children attending the traditional school experienced accelerated BMI gain during summer. Interestingly, even though children attending the year-round school increased engagement in behaviors related to increased risk for overweight and obesity when not attending school during summer vacation, they did exhibit accelerated summer BMI gain. A potential explanation for this may be that children in the year-round school were only exposed to 5 weeks of summer vacation compared to the traditional school children, who experienced 12 weeks away from school. This is consistent with findings from the larger study that found children in the year-round school actually experienced improvements in zBMI during the summer [28]. However, in the larger study the year-round school children experienced accelerated zBMI gains during the traditional school year which largely offset the benefits they experienced during the summer. The reason for year-round school children’s accelerated BMI gain during the school year cannot be explained with the data collected in this study because behaviors were only collected during the spring academic semester and during summer vacation. However, this finding should be replicated and explored in future research.

Findings from this study suggest that providing structured programming during summer vacation may be an efficacious intervention strategy for mitigating accelerated summer BMI gain as children’s behaviors were largely healthier when school was in session. Additionally, children attending the year-round school did not experience accelerated BMI gain during the summer. This is consistent with other studies that have evaluated the impact of providing structured programing on children’s BMI and related behaviors. For instance, a recent single group (i.e., no control group) study examined the impact of a 6-week summer learning program on 20 elementary aged children’s weight status and engagement in obesogenic behaviors during the summer [48]. It showed that children who attended the program had stable weight status over summer vacation and that their obesogenic behaviors were more favorable on days they attended the program than days they did not attend the program. A recent natural experiment examined the impact of attending a 5-week summer school program on 138 adolescent’s body composition over the summer [63]. The study found that children not attending the summer school program showed statistically significantly greater increases in body composition than their counterparts that did not attend summer school. In the absence of structured programming interventions targeting the home (e.g., parent rules related to screens and bed and wake time, social support for physical activity) may be beneficial as well. However, home-based childhood obesity interventions have been mixed [64] and produced minimally effective in the past [65].

This study has a variety of strengths. First, the study collected data continuously for 30+ days during the school year and summer vacation. The volume of data collected on children’s obesogenic behaviors during the school year and summer vacation increases confidence that the data represent children’s habitual engagement in these behaviors. This is also the first study to examine children’s zBMI change in addition to related health behaviors during summer vacation. Further, collecting data on the same children during the school year and summer vacation allows for within-participant comparisons. By implementing a natural experiment and collecting data during the school year, the traditional summer vacation while year-round children attended school, and while all children were on summer vacation, we were able to investigate the causal impact of the school day on children’s obesogenic behaviors. This study was also guided by a theoretical framework, the SDH [6]. Use of theory to guide the generation and testing of hypotheses is widely considered best practice in research and more efficiently moves the field forward [66].

This study also has limitations that should be considered when interpreting the findings. First, only three schools (one following a year-round schedule) were included. Further, the majority of students were Black, and these schools primarily served children from low-income households. Thus, more work with a larger and more representative sample of schools is needed to confirm the findings herein. Second, Fitbit has been shown to have good agreement with polysomnography assessment of sleep and electrocardiography assessment of heartrate, [32, 67, 68] but they have not been used as extensively in sleep and physical activity research. Thus, comparing the sleep and physical activity findings herein to other studies should be done with caution. This study was also conducted in the United States. Thus, these findings may not generalize to other countries around the world. There was also significant participant dropout over the course of the two-year study (> 50%) prompting the recruitment of a refreshment sample in Year 02. It is possible that the drop-outs were systematically different from those participants that remained in the study and/or from the refreshment sample. In order to identify if this was the case baseline demographic and outcome data were examined and no statistically significant differences were found between children who dropped out of the study and those that stayed in the study. Further, the refreshment sample was recruited from the same schools, refreshment children were matched based on age, sex, and race/ethnicity of the children they replaced, and refreshment status was included as a covariate in all statistical models. Another weakness of the current study is the lack of details about children’s days during summer vacation. Based on the SDH children who attended structured programming like summer camps during the summer should not have experienced unhealthy changes in obesogenic behaviors. Future studies should closely track participants’ attendance at structured summer programming. Finally, the measure of foods and drinks consumed in this study focused on snack foods exclusively and was not comprehensive measure of diet. Thus, it may not have been sensitive enough to capture changes in diet from the school year to summer break.

## Conclusions

Children’s sedentary behaviors, screen time, and sleep timing were less favorable during summer vacation compared to the school year and evidence from this study suggests that the school day may regulate unhealthy behaviors and the absence of the school day during summer vacation may cause increases in unhealthy behaviors. These findings are consistent with the SDH which posits that children’s obesogenic behaviors should be worse on relatively less structured days, like summer vacation from school. Sedentary behaviors, screen time, and sleep onset, offset, and midpoint are potential targets for intervention during the summer break.

## Data Availability

Data supporting the results reported in this article are stored at the University of South Carolina. These data will be made available upon request by contacting the first author.

## References

[1] Moreno JP, Johnston CA, Chen TA, O'Connor TA, Hughes SO, Baranowski J (2015). Seasonal variability in weight change during elementary school. Obesity..

[2] von Hippel PT, Workman J (2016). From kindergarten through second grade, US Children's obesity prevalence grows only during summer vacations. Obesity..

[3] Weaver RG, Beets MW, Brazendale K, Brusseau TA. Summer weight gain and fitness loss: causes and potential solutions. Am J Lifestyle Med. 2018;1559827617750576. 10.1177/1559827617750576 PMC6378497 30800015

[4] Franckle R, Adler R, Davison K. Peer reviewed: accelerated weight gain among children during summer versus school year and related racial/ethnic disparities: a systematic review. Prev Chronic Dis. 2014;11. 10.5888/pcd11.130355 PMC4060873 24921899

[5] Watson A, Maher C, Tomkinson GR, Golley R, Fraysse F, Dumuid D (2019). Life on holidays: study protocol for a 3-year longitudinal study tracking changes in children’s fitness and fatness during the in-school versus summer holiday period. BMC Public Health.

[6] Brazendale K, Beets M, Pate RR, Turner-McGrievy B, Kaczynski AT, Weaver RG, et al. Understanding differences between summer vs. school obesogenic behaviors of children: The Structured Days Hypothesis. Int J Behav Nutr Phys Act. 2017:14(1). 10.1186/s12966-017-0555-2 PMC5530518 28747186

[7] Moreno JP, Crowley SJ, Alfano CA, Hannay KM, Thompson D, Baranowski T (2019). Potential circadian and circannual rhythm contributions to the obesity epidemic in elementary school age children. Int J Behav Nutr Phys Act.

[8] Tambalis KD, Panagiotakos DB, Psarra G, Sidossis LS (2018). Insufficient sleep duration is associated with dietary habits, screen time, and obesity in children. J Clin Sleep Med.

[9] Rey-Lopez JP, Vicente-Rodríguez G, Biosca M, Moreno LA (2008). Sedentary behaviour and obesity development in children and adolescents. Nutr Metab Cardiovasc Dis.

[10] Hills AP, Andersen LB, Byrne NM (2011). Physical activity and obesity in children. Br J Sports Med.

[11] Kreitsch KN, Chardon ML, Beebe DW, Janicke DM. Sleep and weight-related factors in youth: a systematic review of recent studies. Sleep Med Rev. 2019. 10.1016/j.smrv.2019.04.010 31100467

[12] Osei-Assibey G, Dick S, Macdiarmid J, Semple S, Reilly JJ, Ellaway A (2012). The influence of the food environment on overweight and obesity in young children: a systematic review. BMJ Open.

[13] Lobstein T, Jackson-Leach R, Moodie ML, Hall KD, Gortmaker SL, Swinburn BA (2015). Child and adolescent obesity: part of a bigger picture. Lancet.

[14] Olds TS, Maher CA, Matricciani L (2011). Sleep duration or bedtime? Exploring the relationship between sleep habits and weight status and activity patterns. Sleep..

[15] Staiano AE, Broyles ST, Katzmarzyk PT (2015). School term vs. school holiday: associations with Children’s physical activity, screen-time, diet and sleep. Int J Environ Res Public Health.

[16] Wing YK, Li SX, Li AM, Zhang J, Kong APS (2009). The effect of weekend and holiday sleep compensation on childhood overweight and obesity. Pediatrics..

[17] Agostini A, Pignata S, Camporeale R, Scott K, Dorrian J, Way A, et al. Changes in growth and sleep across school nights, weekends and a winter holiday period in two Australian schools. Chronobiol Int. 2018:1–14. 10.1080/07420528.2018.1430037 29372811

[18] Wang YC, Vine S, Hsiao A, Rundle A, Goldsmith J (2015). Weight-related behaviors when children are in school versus on summer breaks: does income matter?. J School Health.

[19] Lanningham-Foster L, Foster RC, McCrady SK, Manohar CU, Jensen TB, Mitre NG (2008). Changing the school environment to increase physical activity in children. Obesity..

[20] Nixon GM, Thompson JM, Han DY, Becroft DM, Clark PM, Robinson E (2008). Short sleep duration in middle childhood: risk factors and consequences. Sleep..

[21] McCue MC, Marlatt KL, Sirard J (2013). Examination of changes in youth diet and physical activity over the summer vacation period. Int J Allied Health Sciences and Practice.

[22] Brazendale K, Beets MW, Turner-McGrievy GM, Kaczynski AT, Pate RR, Weaver RG (2018). Children’s obesogenic behaviors during summer versus school: a within-person comparison. J Sch Health.

[23] Atkin AJ, Sharp SJ, Harrison F, Brage S, Van Sluijs EM (2016). Seasonal variation in children’s physical activity and sedentary time. Med Sci Sports Exerc.

[24] Hjorth MF, Chaput J-P, Michaelsen K, Astrup A, Tetens I, Sjödin A (2013). Seasonal variation in objectively measured physical activity, sedentary time, cardio-respiratory fitness and sleep duration among 8–11 year-old Danish children: a repeated-measures study. BMC Public Health.

[25] Zheng C, Huang WY, Wong SH-S (2019). Associations of weather conditions with adolescents’ daily physical activity, sedentary time, and sleep duration. Appl Physiol Nutr Metab.

[26] Tucker P, Gilliland J (2007). The effect of season and weather on physical activity: a systematic review. Public Health.

[27] Kripke DF, Elliott JA, Youngstedt SD, Rex KM (2007). Circadian phase response curves to light in older and young women and men. J Circadian Rhythms.

[28] Weaver RG, Hunt E, Rafferty A, Beets MW, Brazendale K, Turner-McGrievy B, et al. The potential of a year-round school calendar for maintaining Children’s weight status and fitness: preliminary outcomes from a natural experiment. J Sport Health Sci. 2019. 10.1016/j.jshs.2019.05.006 PMC6943754 31921477

[29] Hsieh F, Lavori PW, Cohen HJ, Feussner JR (2003). An overview of variance inflation factors for sample-size calculation. Evaluation & the Health Professions.

[30] Hunt ET, Whitfield ML, Brazendale K, Beets MW, Weaver RG (2019). Examining the impact of a summer learning program on children's weight status and cardiorespiratory fitness: a natural experiment. Eval Program Plann.

[31] Weaver RG, Beets MW, Perry M, Hunt E, Brazendale K, Decker L (2019). Changes in Children’s Sleep and Physical Activity During a One-week versus a Three-week Break from School: A natural experiment. Sleep.

[32] de Zambotti M, Baker FC, Willoughby AR, Godino JG, Wing D, Patrick K (2016). Measures of sleep and cardiac functioning during sleep using a multi-sensory commercially-available wristband in adolescents. Physiol Behav.

[33] Brazendale K, Decker L, Hunt ET, Perry MW, Brazendale AB, Weaver RG (2019). Validity and Wearability of consumer-based fitness trackers in free-living children. Int J Exercise Sci.

[34] Brazendale K, Beets MW, Weaver RG, Perry MW, Tyler EB, Hunt ET (2019). Comparing measures of free-living sleep in school-aged children. Sleep Med.

[35] Tudor-Locke C, Barreira TV, Schuna JM, Mire EF, Chaput J-P, Fogelholm M (2015). Improving wear time compliance with a 24-hour waist-worn accelerometer protocol in the international study of childhood obesity, lifestyle and the environment (ISCOLE). Int J Behav Nutr Phys Act.

[36] Carissimi A, Dresch F, Martins AC, Levandovski RM, Adan A, Natale V (2016). The influence of school time on sleep patterns of children and adolescents. Sleep Med.

[37] Bei B, Wiley JF, Trinder J, Manber R (2016). Beyond the mean: a systematic review on the correlates of daily intraindividual variability of sleep/wake patterns. Sleep Med Rev.

[38] Acebo C, Sadeh A, Seifer R, Tzischinsky O, Wolfson AR, Hafer A (1999). Estimating sleep patterns with activity monitoring in children and adolescents: how many nights are necessary for reliable measures?. Sleep..

[39] Welk GJ, Corbin CB (1995). The validity of the Tritrac-R3D activity monitor for the assessment of physical activity in children. Res Q Exerc Sport.

[40] Sallis JF, Buono MJ, Roby JJ, Carlson D, Nelson JA (1990). The Caltrac accelerometer as a physical activity monitor for school-age children. Med Sci Sports Exerc.

[41] Janz KF. Validation of the CSA accelerometer for assessing children's physical activity. Med Sci Sports Exerc. 1994. 8183103

[42] Simons-Morton BG, Taylor WC, Huang IW (1994). Validity of the physical activity interview and Caltrac with preadolescent children. Res Q Exerc Sport.

[43] Epstein LH, Paluch RA, Kalakanis LE, Goldfield GS, Cerny FJ, Roemmich JN (2001). How much activity do youth get? A quantitative review of heart-rate measured activity. Pediatrics.

[44] Chandler J, Brazendale K, Beets M, Mealing B. Classification of physical activity intensities using a wrist-worn accelerometer in 8–12-year-old children. Pediatric Obesity. 2015. 10.1111/ijpo.12033 25893950

[45] Gavarry O, Bernard T, Giacomoni M, Seymat M, Euzet J, Falgairette G (1997). Continuous heart rate monitoring over 1 week in teenagers aged 11–16 years. Eur J Appl Physiol Occup Physiol.

[46] Eisenmann JC, Bartee RT, Wang MQ (2002). Physical activity, TV viewing, and weight in US youth: 1999 youth risk behavior survey. Obesity..

[47] Tandon PS, Zhou C, Sallis JF, Cain KL, Frank LD, Saelens BE (2012). Home environment relationships with children’s physical activity, sedentary time, and screen time by socioeconomic status. Int J Behav Nutr Phys Act.

[48] Hunt E, Whitfield M, Brazendale K, Beets MW, Weaver RG. Examining the impact of a summer learning program on Children's weight status and cardiorespiratory fitness. J Community Health. in press. 10.1016/j.evalprogplan.2019.02.009 PMC8087453 30939299

[49] Neuhouser ML, Lilley S, Lund A, Johnson DB (2009). Development and validation of a beverage and snack questionnaire for use in evaluation of school nutrition policies. J Am Diet Assoc.

[50] Kasper N, Mandell C, Ball S, Miller AL, Lumeng J, Peterson KE (2016). The healthy meal index: a tool for measuring the healthfulness of meals served to children. Appetite..

[51] Kuczmarski R, Ogden C, Guo S, Grummer-Strawn L, Flegal K, Mei Z (2002). CDC growth charts for the US: methods and development. Vital Health Stat.

[52] Benjamini Y, Hochberg Y (1995). Controlling the false discovery rate: a practical and powerful approach to multiple testing. J R Stat Soc Ser B Methodol.

[53] Stoner L, Castro N, Signal L, Skidmore P, Faulkner J, Lark S, et al. Sleep and adiposity in preadolescent children: the importance of social jetlag. Child Obes. 2018. 10.1089/chi.2017.0272 29298086

[54] Golley RK, Maher CA, Matricciani L, Olds TS (2013). Sleep duration or bedtime? Exploring the association between sleep timing behaviour, diet and BMI in children and adolescents. Int J Obesity (2005).

[55] Jarrin DC, McGrath JJ, Drake CL (2013). Beyond sleep duration: distinct sleep dimensions are associated with obesity in children and adolescents. Int J Obes.

[56] Arora T, Taheri S (2015). Associations among late chronotype, body mass index and dietary behaviors in young adolescents. Int J Obes.

[57] Adamo KB, Wilson S, Belanger K, Chaput J-P (2013). Later bedtime is associated with greater daily energy intake and screen time in obese adolescents independent of sleep duration. J Sleep Disord Ther.

[58] Okada C, Tabuchi T, Iso H. Association between skipping breakfast in parents and children and childhood overweight/obesity among children: a nationwide 10.5-year prospective study in Japan. Int J Obesity. 2018;1. 10.1038/s41366-018-0066-5 29686380

[59] Keski-Rahkonen A, Kaprio J, Rissanen A, Virkkunen M, Rose RJ (2003). Breakfast skipping and health-compromising behaviors in adolescents and adults. Eur J Clin Nutr.

[60] Zerón-Rugerio MF, Hernáez Á, Porras-Loaiza AP, Cambras T, Izquierdo-Pulido M (2019). Eating jet lag: a marker of the variability in meal timing and its association with body mass index. Nutrients..

[61] Tanskey LA, Goldberg JP, Chui K, Must A, Sacheck JM (2019). Accelerated summer weight gain in a low-income, Ethnically Diverse Sample of Elementary School Children in Massachusetts. Childhood Obesity.

[62] Burrows TL, Martin RJ, Collins CE (2010). A systematic review of the validity of dietary assessment methods in children when compared with the method of doubly labeled water. J Am Diet Assoc.

[63] Park K-S, Lee M-G (2015). Effects of summer school participation and psychosocial outcomes on changes in body composition and physical fitness during summer break. J Exerc Nutr Biochem.

[64] Pamungkas RA, Chamroonsawasdi K (2019). Home-based interventions to treat and prevent childhood obesity: a systematic review and meta-analysis. Behav Sciences.

[65] Showell NN, Fawole O, Segal J, Wilson RF, Cheskin LJ, Bleich SN (2013). A systematic review of home-based childhood obesity prevention studies. Pediatrics..

[66] Painter JE, Borba CP, Hynes M, Mays D, Glanz K (2008). The use of theory in health behavior research from 2000 to 2005: a systematic review. Ann Behav Med.

[67] Liang Z, Martell MAC. Validity of consumer activity wristbands and wearable EEG for measuring overall sleep parameters and sleep structure in free-living conditions. Journal of Healthcare Informatics Research. 2018:1–27. 10.1007/s41666-018-0013-1 PMC8982823 35415400

[68] de Zambotti M, Goldstone A, Claudatos S, Colrain IM, Baker FC (2018). A validation study of Fitbit charge 2™ compared with polysomnography in adults. Chronobiol Int.

